# Development of Thermosensitive Hydrogels with Tailor-Made Geometries to Modulate Cell Harvesting of Non-Flat Cell Cultures

**DOI:** 10.3390/gels10120802

**Published:** 2024-12-06

**Authors:** Rubén García-Sobrino, Enrique Martínez-Campos, Daniel Marcos-Ríos, Zenen Zepeda-Rodríguez, Juan L. Valentín, Raúl Sanz-Horta, Marina León-Calero, Helmut Reinecke, Carlos Elvira, Alberto Gallardo, Juan Rodríguez-Hernández

**Affiliations:** 1 Polymer Functionalization Group, Departamento de Química Macromolecular Aplicada, Instituto de Ciencia y Tecnología de Polímeros-Consejo Superior de Investigaciones Científicas (ICTP-CSIC), Calle Juan de la Cierva, n° 3, 28006 Madrid, Spain; emartinezcampos79@gmail.com (E.M.-C.); danielmarcos14@gmail.com (D.M.-R.); r.sanzhorta@gmail.com (R.S.-H.); marina.3dp@ictp.csic.es (M.L.-C.); icthr14@ictp.csic.es (H.R.); celvira@ictp.csic.es (C.E.); gallardo@ictp.csic.es (A.G.); 2 Group of Organic Synthesis and Bioevaluation, Instituto Pluridisciplinar, Universidad Complutense de Madrid (UCM), Associated Unit to the ICTP-IQM-CSIC, Paseo Juan XXIII, n° 1, 28040 Madrid, Spain; 3 Department of Applied Mathematics, Materials Science and Engineering and Electronic Technology, Universidad Rey Juan Carlos, Calle Tulipán s/n, 28933 Móstoles, Spain; 4 Elastomers Group, Instituto de Ciencia y Tecnología de Polímeros-Consejo Superior de Investigaciones Científicas (ICTP-CSIC), Calle Juan de la Cierva, n° 3, 28006 Madrid, Spain; zenen.zepeda90@gmail.com (Z.Z.-R.); jlvalentin@ictp.csic.es (J.L.V.)

**Keywords:** biomaterials, smart hydrogel, 3D-printing, sacrificial mold, cell sheet engineering

## Abstract

Considering the complexity in terms of design that characterizes the different tissues of the human body, it is necessary to study and develop more precise therapies. In this sense, this article presents the possibility of fabricating photocurable thermosensitive hydrogels with free geometry and based on N-Vinyl Caprolactam (VCL) with the aim of modulating the adhesion of non-planar cell cultures. The fabrication process is based on the use as a mold of two-layer thick water-soluble polyvinyl alcohol (PVA) previously printed by Extrusion Material (MatEx). From this technology it has been possible to obtain hydrogels with different 3D geometries and different crosslinking percentages (2, 4 and 6 mol%). Studies have shown that networks reduce their thermosensitivity not only when the percentage of crosslinking in the formulation increases, but also when the thickness of the hydrogel obtained increases. Based on this reduction in thermosensitivity, the less crosslinked (2 mol%) hydrogels have been evaluated to carry out a novel direct application in which hydrogels with curved geometry have allowed cell adhesion and proliferation at 37 °C with the endothelial cell line C166-GFP; likewise, non-aggressive cell detachment was observed when the hydrogel temperature was reduced to values of 20 °C. Therefore, the present manuscript shows a novel application for the synthesis of free-form thermosensitive hydrogels that allows modulation of non-planar cell cultures.

## 1. Introduction

Hydrogels have become some of the most extensively used materials in the biomedical sector over the last few decades for a myriad of applications including scaffolds in tissue engineering, drug delivery, biosensors, wound healing dressings or self-healing, to mention just a few [1,2,3,4,5,6,7]. These hydrophilic polymers are crosslinked systems that swell in the presence of aqueous media, a characteristic that gives them a high resemblance to living tissues, thus reducing possible rejections in tissue engineering and regenerative medicine (TERM) studies [8]. It should be noted that, being commonly reported simple hydrogel systems such as homogeneous films, living tissues belonging to the human body often present structures with complex characteristics such as, for example, anisotropic and/or hierarchical properties [9,10,11]. In this sense, processes to obtain hydrogels with complex structural characteristics that bring their properties closer to those of the native tissue represent an improvement over conventional process. So far, although the fabrication of precise 3D hydrogels is still a great challenge for the scientific community, numerous techniques have been developed in recent years that allow for the production of increasingly complex structural networks at the nano-, micro- and macro-scales [12].

In this context, 3D printing and advances in bio-printing could favor the formation of structures with complex geometries that would increase the scope of application of the aforementioned hydrogels [13]. However, despite the improvements developed in 3D printing in fields such as tissue engineering, the high-water absorption capacity of hydrogels drastically reduces their mechanical capacity and therefore hinders their printability with defined and customized geometries [14].

In terms of obtaining hydrogels with complex shapes and structures, manufacturing techniques using 3D printed sacrificial molds (instead of 3D printing the hydrogel itself) have proven to be an attractive option [13]. Briefly, this technique is based on the printing of complex designs that act as molds for subsequent filling of the biomaterial protagonist. Then, using different methods (physical or chemical) the mold is removed and the biomaterial is obtained with the final designed geometry. In this way, materials with limited printing options, such as highly swellable hydrogels, can be obtained using complex structures as support that only 3D printing technologies can achieve. One of the conditions required for sacrificial material, in addition to allowing fidelity in terms of complex shapes, is that it shows ease and biotolerance in terms of disposal, avoiding possible contamination of the subsequent target culture.

Following the classification proposed by Wang and Zhou [14], sacrificial element removal methods can be divided into chemical or physical depending on the phenomenon involved in the mold removal process. In the case of the first one, and in a generic way, it usually allows for obtaining structures with better resolution but requires more expensive and complex methods such as inverse crosslinking methods (for instance using alginate as a sacrificial element) or photo-crosslinking processes [15,16,17]. On the other hand, concerning physical methods, such as those developed in this work, they are usually developed with lower cost methods based on demolding methods comprising dissolution of the printed mold (use of molds such as high impact polystyrene and poly vinyl alcohol, HIPS and PVA, respectively; and their related solvents such as d-limonene and water) and/or from temperature variations when using, for instance, thermo-reversible materials such as gelatine and Pluronic F127 [18,19,20,21]. In short, although in general with lower resolution and shape fidelity, the processes of elimination of the sacrificial element by physical methods are preferred when it is necessary to carry out subsequent biological studies as the reaction does not involve the use of chemical agents that could compromise its cytocompatibility [13].

In this work, we propose to explore the use of 3D printed PVA molds to obtain thermosensitive VCL-based hydrogels by photocuring precursor formulations. These hydrogels, in their flat form, have previously exhibited excellent mechanical properties and thermosensitivity. The term thermosensitive refers to the capacity of this family of hydrogels to drastically modify the architecture of the network depending on changes in the external temperature, causing variations in terms of swelling and thus modifications in terms of mechanics, transmittance or even permeability of the network [22]. In addition, these flat hydrogels with the possibility of being functionalized by the inclusion of different monofunctional methacrylates demonstrate cytocompatibility, supporting cell adhesion and proliferation at physiological temperature or 37 °C, while, when the temperature is reduced to values below a transition value (approximately 34 °C for this family of hydrogels), the systems allow a non-aggressive detachment of the cell culture obtained, keeping the cultured cell interactions intact [23,24,25,26,27]. This phenomenon is based on changes in terms of the hydrophobicity of the macromolecule involved, which, at temperatures above a certain transition value, presents preferential hydrophobic behavior, being hydrophilic below it and thus allowing the entry of a medium into the systems that favors the detachment of the culture described. This technology belongs to the cell sheet engineering field, which was described first in 1990 by Okano’s group [28]. They took advantage of the thermosensitivity of the polymer poly-N-isopropylacrylamide (pNIPAAm) to modulate cell adhesion and over the years have allowed for its application in the clinical phase in regeneration therapies for lesions such as myocardial ischemia, corneal epithelium, or periodontal ligament, among others [29]. Although pNIPAAm has garnered the most attention as a thermosensitive polymer for biomedical applications to date [30], the pVCL polymer worked on in this manuscript possesses similar properties that make it a very attractive polymer for applications associated with this sector as well. For example, both polymers are water-soluble, non-ionizable and exhibit a similar LCST transition temperature close to the physiological range (32–34 °C) [31]. However, the key to the use of VCL as a precursor derives from previous efforts developed by the research group to obtain pseudo-ordered polymeric networks with excellent mechanical properties as already mentioned (169 ± 6 KPa is the value of the elastic modulus of hydrated hydrogels at 20 °C in compression studies) [32]. The term pseudo-ordered refers to obtaining hydrogels in a single step based on the difference in reactivity of the precursors involved [24,33].

The present work has addressed the preparation of the cited VCL-based thermosensitive hydrogels in complex 3D shapes and the preparation of fully customizable, cytocompatible, highly resistant, thermoresponsive hydrogels adaptable to any complex geometry that could be very attractive for repairing certain lesions. Despite numerous advances in this technology, and to the best of our knowledge, all thermosensitive cell manipulation supports found in the literature are based on a coating technology that allows cell manipulation of flat cultures only [34,35,36]. In this sense, no group has proposed the manipulation of non-planar cultures with smart support. In an effort to bring cell sheet detachment technology closer to complex structures, our group recently described the preparation of the aforementioned hydrogels with a cylindrical shape using PP molds [32]. It is necessary to highlight the dimensional complexity and the high requirements involved in the regeneration of the different damaged tissues present in the human body. This is why it is essential to search for increasingly versatile and precise technologies. In the case of the tubular structures described above, the process was limited by the PP molds used. Thus, based on the technology of obtaining hydrogels with complex geometries using printed sacrificial molds, we propose to obtain hydrogels with complex geometry that will allow us to reduce the design limitations exposed. With the technology proposed in this manuscript, a versatility of design would be achieved that would allow the precise development of tailor-made cell therapies.

PVA is a first-choice material to be used as a sacrificial mold in the preparation of biomaterials as it is printable, non-toxic and water-soluble. There are several examples of its use for the preparation of hydrogels [37,38,39]. Its sacrificial character makes it more interesting than non-sacrificial molds, such as those previously described by us to prepare tubular thermosensitive hydrogels and tubular cell monolayers able to detach upon temperature decrease [32], because it is possible to design and use mold structures interpenetrated with the final material. However, PVA-based printed materials are not completely transparent and therefore there are very few uses in processes that require irradiation. B. Pan et al. [38] used it to crosslink gelatin-MA, but to the best of our knowledge it has not been used for the preparation of molds to be employed for photopolymerization processes, which is the method we will describe in this work.

Therefore, based on the above-mentioned strategy, this work presents the optimization of the synthesis process through the photocuring of thermosensitive hydrogels using water-soluble PVA sacrificial templates printed by Material Extrusion (MatEx) 3D technology. After this, an exhaustive study of the influence of the geometry, thickness and crosslinking of non-planar systems proposed on their thermosensitivity was carried out. Finally, and after understanding the thermosensitive behavior of the networks, hydrogels with curved geometries prepared using sacrificial printed templates were evaluated as smart supports for advanced and customizable cell therapies.

## 2. Results and Discussion

**Figure 1:**
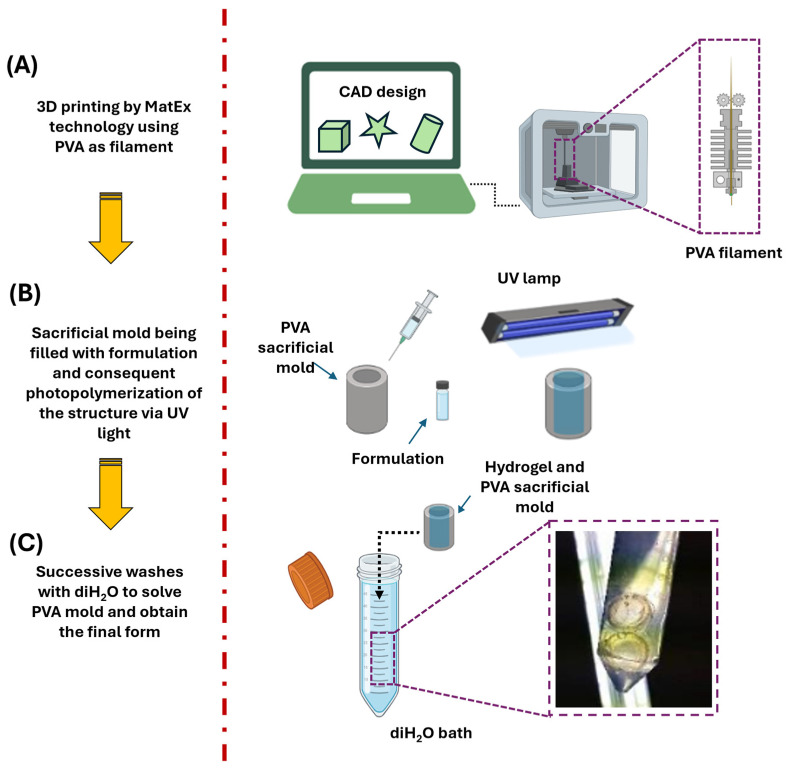
Schematic summary of the steps required to fabricate three-dimensional hydrogels with complex shapes from water-soluble molds (PVA) printed by MatEx technology: (**A**) Design and printing of PVA sacrificial mold. (**B**) Preparation of the formulation and filling in the mold with subsequent light-curing by external UV lamp. (**C**) Finally, washing with water allowed us to remove PVA and residual formulation to obtain the final geometry.

The hypothesis put forward in this work is that from the use of PVA sacrificial molds prepared by 3D printing (MatEx), photocured hydrogels with complex geometries can be obtained, which may be useful for different objectives in the field of tissue engineering. Briefly, (see the scheme in Figure 1) after printing the PVA sacrificial mold (that will define the final shape of the target hydrogel), the light-curing formulation is injected into the mold. With the exposure to UV light, the photopolymerization of the elements is generated, and after a subsequent washing step with water, and due to the solubility of PVA in water, allows the hydrogel to be obtained in its final shape.

In this work we aim to develop a proof of concept using light-curable formulations based on the T-sensitive precursor, vinyl-caprolactam (VCL). These hydrogels were prepared using a mixture of two different crosslinkers (EGDMA and DVI). Previous studies focusing on the influence of this EGDMA/DVI ratio on the properties of hydrogels showed that EGDMA-rich hydrogels were more robust, but less transparent than DVI-rich ones [32]. In this manuscript, an EGDMA/DVI molar ratio of 80/20 has been selected, which allows for obtaining networks not completely transparent at 37 °C, but with a high degree of robustness, which is of great interest in the present study of hydrogels with complex shapes since they must maintain their shape during the initial swelling. On the other hand, the presence of EtOH in the formulation favors homogeneity in terms of filling by reducing the viscosity of the mixture.

### 2.1. Optimization of the Printing Process of Sacrificial PVA Mold

**Figure 2:**
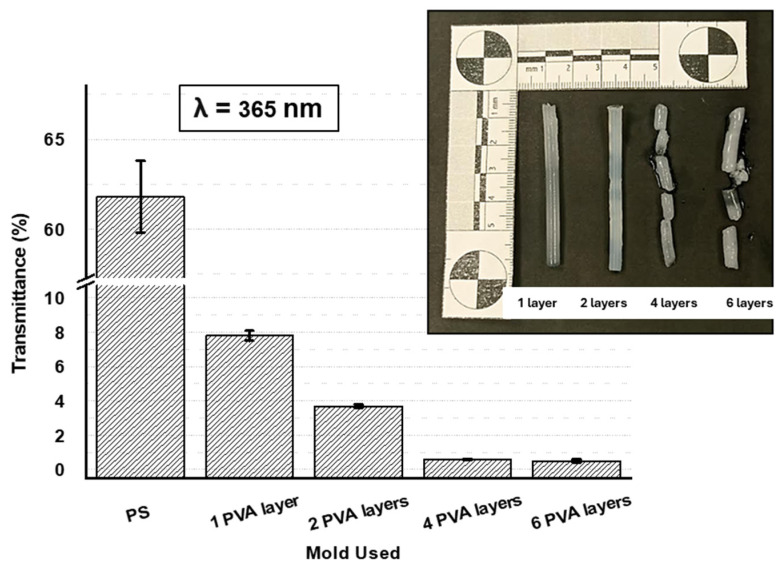
Images of the cylinder’s hydrogels obtained depending on the number of PVA mold layers and transmittance percentage measured at 365 nm (wavelength used for photopolymerization of T-sensitive pVCL hydrogels) of the templates made by PVA as a function of the number of layers compared to a control template.

To address the proposed hypothesis, it is necessary to provide evidence that PVA can be used as a template for light-curing processes, i.e., that it should allow the passage of UV light and thus enable the photopolymerization step to occur. At first glance, while PVA printed pieces with very few layers are translucent, thick pieces are opaque. For this reason, tubular PVA molds with 3 mm of inner diameter and different numbers of layers were prepared to evaluate their potential as molds for photopolymerization. The number of layers of the mold contour were one, two, four and six layers (which are equivalent to 0.4, 0.8, 1.6 and 2.4 mm wall thickness, respectively). Single-layer PVA molds presented resin leaking issues, although this apparently allows for optimal construction, because the injection of the formulation to be light-cured is lost due to the gaps left by the imperfect printing between the deposited layers (see Figure 2). Therefore, according to our findings the minimum number of layers needed to prevent formulation leaks during injection is two. An increase in the number of layers up to four and six, leads to hydrogels with an ill-defined final structure most probably due to the limited UV light penetration. In this sense, therefore, the preparation of 3D printed parts with two contour layers resulted in the method with the lowest material cost while maintaining the structure without resin leakage.

In order to relate the previous results with the light intensity going through the PVA walls and, thus, the effective curing of the formulation inside the mold, the transparency of PVA has been studied as a function of the number of layers at the wavelength used for photopolymerization (365 nm). Also in Figure 2, the transmittance of PVA molds with walls made from one up to six layers was analyzed and compared to a PS mold covered with an LDPE sheet (named as PS mold throughout the manuscript), which has been previously used to efficiently prepare 2D film hydrogels [23,24,25,26,27]. In the case of PVA as a mold, the printed molds show lower transparency compared to a traditional PS mold (reduction of more than 85%), with the four and six layer molds being almost opaque in the analyzed wavelength with values below 1%. This observation clearly evidenced that photopolymerization does not occur or occurs only to a limited extent in those cases where molds with four up to six layers of PVA have been employed.

Despite the dramatic reduction of UV light passing through the mold walls (close to 4%) in comparison with control PS molds, molds printed with two layers of PVA allow the fabrication of hydrogels inside the molds. However, a larger exposure time is required for the crosslinking process (40 min) allowing to obtain networks with optimal properties despite the low transparency of the mold. To ensure that the systems manufactured by the new process maintain identical characteristics as those hydrogels manufactured by conventional method, flat molds with PVA sheets printed with two layers were prepared to obtain hydrogel films with a thickness of 0.5 mm. The hydrogels prepared using this strategy were compared to the hydrogel with the same thickness obtained using PS sheets as mold using FTIR spectroscopy and swelling analysis. As shown in Figure S1 the FTIR spectra in both cases are identical. In addition, the swelling of the systems in PBS, measured at a temperature of 10 °C, is also rather similar independently of the type of material employed for the mold (being S, 6.5 ± 0.1 and 6.7 ± 0.2 for the cases of PS and PVA molds, respectively). Therefore, hydrogels manufactured using sacrificial molds comprising two layers of PVA will be used for the following experiments.

### 2.2. 2D Planar Hydrogels

Thermosensitive hydrogels such as those prepared in this work are systems that, when swollen in water, have variable volume and dimensions dependent on temperature [40]. Moreover, these volumes and dimensions are different from those of fresh hydrogels obtained just after photocuring. It is of interest to design the mold dimensions taking into account the target dimensions at different temperatures. It should also be considered that the dimensional differences between water-swollen and fresh hydrogels can be modulated by varying the crosslink density, since the higher the crosslink density, the lower the water swelling. This aspect has been addressed in this work using the flat-shaped (2D) hydrogels described in the previous paragraph as control hydrogels to study swellings. In addition, the influence of the hydrogel thickness has been included in the study, since 3D parts can have very complex geometries. This study has been complemented with a theoretical analysis of dimensional variations for each swelling.

In order to know the influence of the crosslinking percentage on the final quality of the pieces, three formulations have been proposed throughout the manuscript. The crosslinking molar percentages proposed were 2, 4 and 6 mol%, which have been labelled throughout the article as HYD-2, 4 and 6, respectively. Likewise, to favor the homogeneity of the study, the results for HYD-2, 4 and 6, will be represented by black, red and blue, respectively. To structurally confirm the incorporation of crosslinkers, flat networks with 0.5 mm of thickness for the different crosslinkers contents were prepared as indicated before and characterized by FTIR-ATR spectroscopy (see Figure S2). Figure S2 shows that the intensity of the peak located at 1720 cm^−1^, which is assigned to the ester vibration of majority crosslinker EGDMA [41], increases with the crosslinker content, thus evidencing its gradual incorporation as a function of the feed content.

**Figure 3:**
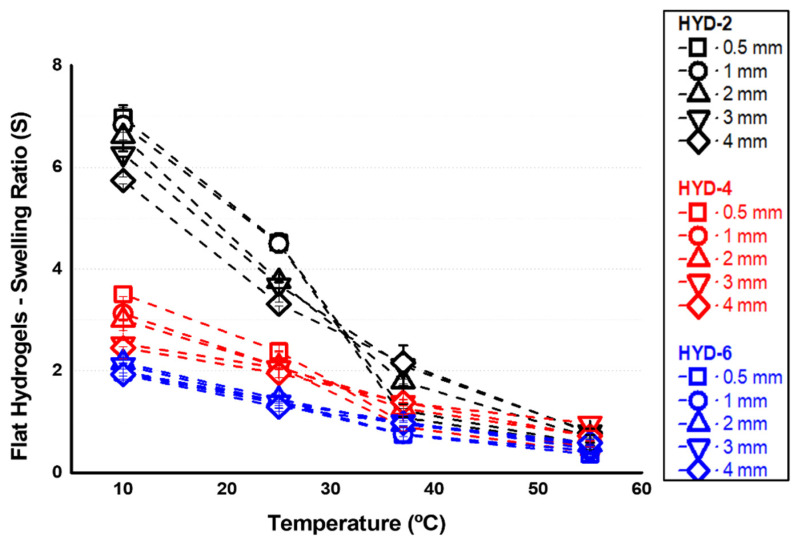
Swelling ratio in PBS medium of flat hydrogels with different crosslinker degrees (HYD-2, 4 and 6) and thickness (0.5, 1, 2, 3 and 4 mm).

Once it was confirmed that the crosslinkers were adequately incorporated into the network, the systems were prepared and evaluated. For each of the three crosslinker contents explored, systems with fresh thicknesses (internal dimensions of the PVA mold) of 0.5, 1, 2, 2, 3, and 4 mm were prepared. For all these hydrogels, equilibrium swelling in water was determined gravimetrically for representative temperatures (10, 25, 37 and 55 °C, see Figure 3). The transition temperature value VPTT (Volume Phase Transition Temperature) of these hydrogels is in the range of 33–35 °C, according to previous studies [32]. The thermosensitivity in water of VCL-based systems is based on a modification with temperature of the balance between polymer–polymer and polymer–water interaction, as this balance is displaced towards the second type of interaction below the transition temperature and thus allowing greater absorption of the medium [22]. Based on the described, and as can be seen in Figure 3, the thermosensitivity of the networks in terms of swelling can be observed. At values above the transition value, the hydrogel releases part of the absorbed medium as a result of a preferential interaction between the polymer chains with respect to the surrounding medium. In the same way, Figure 3 also shows an important influence of the degree of crosslinking on the swelling, especially below the VPTT, as well as on the extent of thermosensitivity, i.e., on the swelling variations when crossing the VPTT. This is an expected result since an increase in the crosslinking density reduces the expansion capacity of the network, this reduction being more important at low temperatures where the network is more hydrophilic and absorbs more water. It is important to note that all systems are in a hydrophobic state at 37 °C, confirming that the value of the transition interval, VPTT, is below the physiological range, ideal for the objective of using these platforms for cell sheet detachment technologies. Figure 3 also shows a slight influence of the thickness, especially for the less crosslinked systems. Despite being a homogeneous chemical structure, as the thickness of the systems increases, their thermosensitivity and swelling capacity slightly decrease.

Regarding the dimensional variations with respect to the fresh material defined by the inner mold dimensions, the precursor formulations of the hydrogels prepared in this work contain ethanol. If we consider that the photocuring conversion is 100%, the fresh hydrogel has an ethanol content in terms of m_EtOH_/m_POL_ weight ratio of 0.35 (being POL the polymer fraction), since the ethanol density is 0.79 g/mL. The volume ratio, assuming that the density of the polymeric network is 1.3 g/mL, is 0.58 (V_EtOH_/V_POL_). A hydrogel swollen in water at equilibrium has a mass ratio of m_H2O_/m_POL_ defined by the equilibrium swelling value S_eq_. The volume ratio V_H2O_/V_POL_ would be S_eq_/1.3. Therefore, the volume ratio of the swollen material at equilibrium to that of the fresh material (F) is calculated following Equation (1):

(1) $$\frac{V_{eq}}{V_{F}}=\frac{\left(1+\frac{S_{eq}}{1.3}\right)}{1.58}.$$

Considering an isotropic swelling, based on previous studies [32], a given linear dimension A will have the variation (between the dimension of the fresh material after photocuring and the swollen material at equilibrium) of

(2) $$\frac{A_{eq}}{A_{F}}=\sqrt[{3}]{\frac{V_{eq}}{V_{F}}}.$$

**Figure 4:**
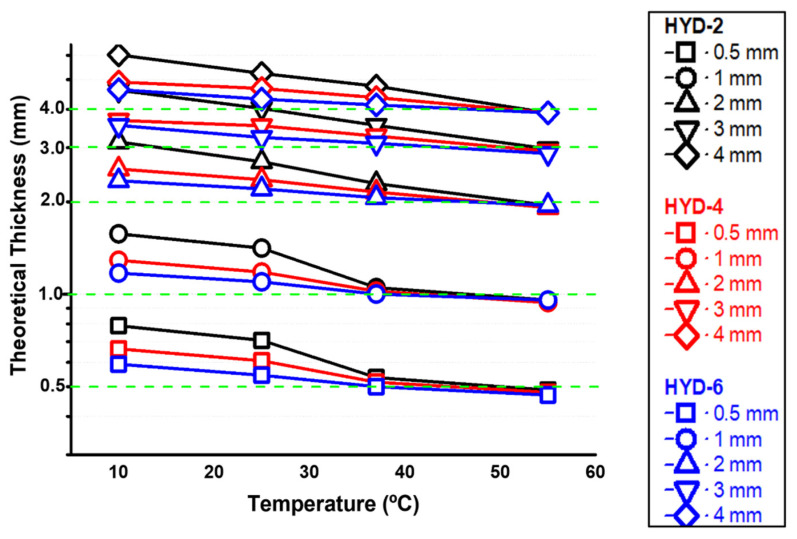
Theoretical description of swelling vs. temperature of flat systems as a function of thickness and degree of crosslinking.

These theoretical calculations have been represented in Figure 4 where, using Equation (2), the calculated thicknesses for the experimentally obtained swellings are listed. It can be seen that, depending on the degree of crosslinking and the temperature, the hydrogel dimensions are higher, closer, or lower than those of the fresh material, i.e., those of the mold. This theoretical prediction can be useful to properly choose the dimensions of the mold according to the target dimensions for a given temperature.

**Figure 5:**
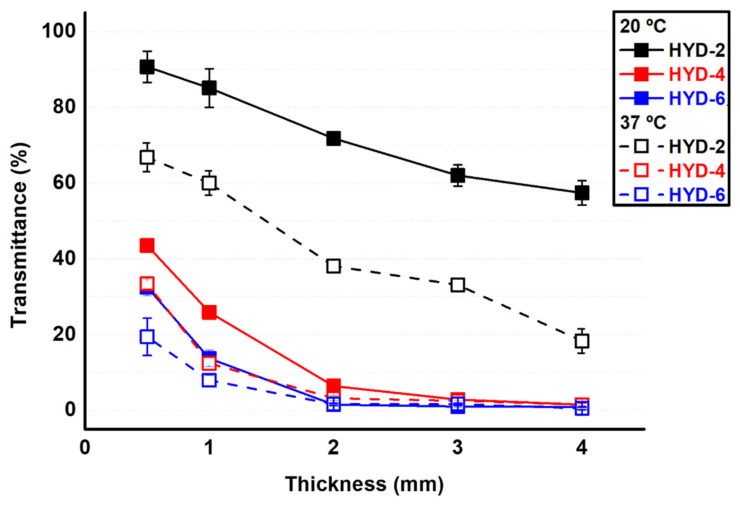
Transmittance percentage in PBS of hydrogels (HYD-2, 4 and 6) as a function of hydrogel thickness for two temperatures (20 and 37 °C).

Similarly, the proposed flat systems were studied under spectrophotometry in PBS and in the visible range (600 nm) in order to analyze the effect of thickness and crosslinking percentage on the optical properties. With reference to transmittance, it is directly related to the structural distribution of the network [42,43]. Figure 5 shows the transmittance values for two temperatures, 20 and 37 °C, i.e., below and above the transition value. As with the gravimetry study, the grids show differences in terms of transmittance as a function of temperature, denoting the thermosensitivity of the systems. It can be observed how the transmittance decreases when reaching the hydrophobic range (37 °C) due to the fact that the system releases part of the water when it exceeds the transition temperature. It undergoes a reordering of the polymeric chains (remember that in this temperature range the chains involved, with hydrophobic character, show a preference between them), folding among themselves and thus reducing the light path [22]. This difference in opacity decreases with an increase in the percentage of crosslinking in the formulation. Equally, an increasing amount of EGDMA (crosslinker majority) also reduced the transparency as has already been described in the case of pVCL hydrogels developed by our group [32]. Based on what has been described, samples HYD-4 and 6 show high opacity, while HYD-2 shows high transmittance values at temperatures below the transition value regardless of the quantified thickness. However, at physiological temperature, HYD-2 samples show some opacity at thicknesses equal to or greater than 2 mm, which would make it difficult to perform cell monitoring studies on their surface in in vitro studies. In this sense and taking into account that the transparency of the samples also decreases as the thickness of the obtained hydrogel increases, it seems that only HYD-2 up to 2 mm in thickness shows transparency values (close to a value of 40%) that could enable cellular monitoring.

It is important to note that the surfaces of the hydrogels manufactured by this technology presented an intrinsic roughness based on the slice generated by the 3D printing itself. Additive manufacturing and especially MatEx technology [44] exhibit roughness along the *z*-axis, the 200 µm layer height in this case, which leads to a wrinkle-like pattern on the PVA surface, with a wrinkling period (W_P_) of around 198 ± 9 µm and a wrinkling amplitude (W_A_) of 264 ± 11 µm. This mold structure will thus be transferred to the hydrogel as a negative pattern (see basic formation scheme above Table 1). Similarly, in the mentioned table the roughness data results at two temperatures (20 and 55 °C) are summarized. Associated studies in which wrinkle size could be regulated could play an additional role in cellular processes such as cell adhesion, proliferation and differentiation [45,46]. Concerning thermosensitivity, Table 1 also shows thermosensitivity in terms of wrinkle variation as a function of temperature for all hydrogels tested, again with decreasing thermosensitivity as the crosslinking of the system increases. Finally, it should be noted that all 3D profilometry figures have been included as Supplementary Figure S3.

### 2.3. Non-Planar Hydrogels

In order to study the feasibility of the process (use of two-layer printed PVA as a sacrificial mold for photocuring hydrogel formulations) for the preparation of non-planar hydrogels, i.e., fabricating different geometric shapes, in a first step molds and hydrogels with different basic designs as well as cylinders and spheres with different diameters were prepared. As will be described later, in a second step, other advanced structures such as tubes and scaffolds of different sizes were equally designed and prepared. It is worth noting that photopolymerization techniques are commonly used for polymerization processes, involving the synthesis of polymers of limited thickness [47,48], so it is not possible to obtain thick parts using this approximation. Therefore, throughout this point, different thicknesses will be analyzed to establish the limits of the technology proposed for light-curing VCL networks. These systems are prepared for the three crosslinking grades HYD-2, HYD-4 and HYD-6.

#### 2.3.1. Cylinders and Spheres

For each of the HYD-2, HYD-4 and HYD-6 formulations, solid tubes of 2, 4, 6 and 8 mm in diameter (12 tube types in total), as well as solid spheres of 6, 8 and 10 mm in diameter (nine sphere types) were prepared (see Table 2). All samples were properly photocured, resulting in well-defined hydrogels after dissolution in water of the PVA sacrificial mold. Table 1 shows photographs of each type of sample swollen in water. It can be observed that, for both cylinders and spheres, the diameter and opacity increase with an increase in the degree of crosslinking. The aforementioned surface structuring is also observed, the negative of the structuring associated with the layer size in the printing of the PVA mold.

**Figure 6:**
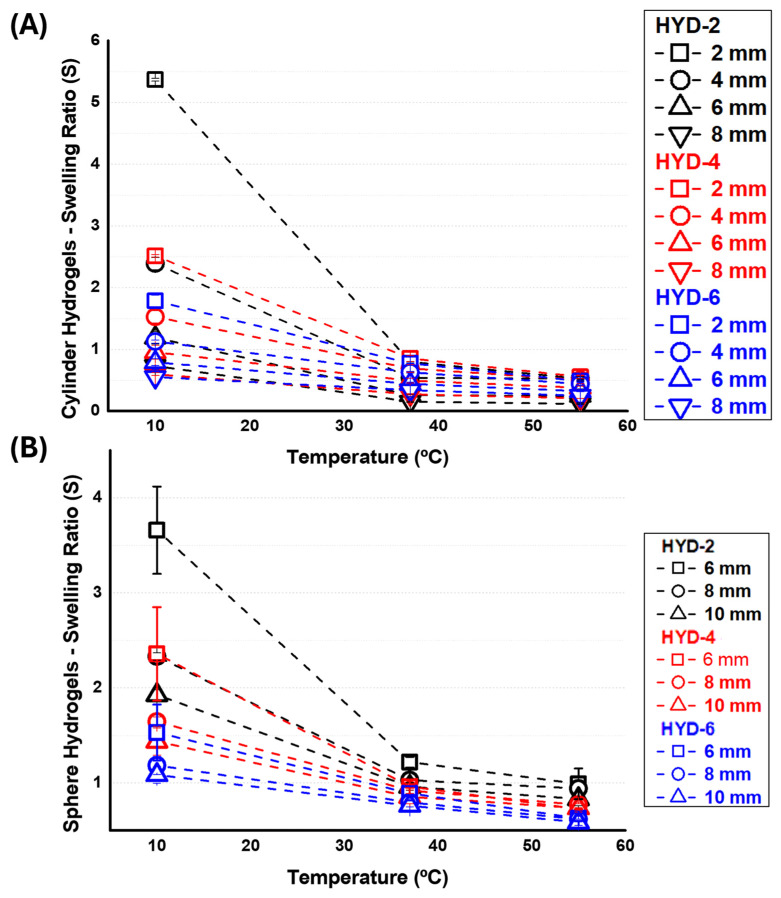
Swelling ratio in PBS at different of: (**A**) cylinders with different diameters (2, 4, 6 and 8 mm) and (**B**) spheres with different diameters (6, 8 and 10 mm) for three representative temperatures: 10, 37 and 55 °C.

The swelling in water of the different samples was evaluated gravimetrically as a function of temperature for three representative values (10, 37 and 55 °C, see Figure 6). Variations in terms of swelling were observed throughout the range of temperatures analyzed, thus evidencing the thermosensitivity of the hydrogels. This characteristic is, as in the previous case of flat hydrogels, dependent on the crosslinking percentage, so that when the number of knots in the network increases, the absorption capacity of the cylindrical systems is reduced.

On the other hand, it can be observed that both cylinders and spheres swell significantly less than flat films. However, it is not possible to state a clear influence of geometry without taking into account the dimensions; the swelling capacity of the networks also decreases as the diameter or thickness increases, especially in cylinders and spheres. In this sense, the films are not completely comparable systems as their thickness is smaller than the diameter dimensions of both cylinders and spheres.

**Figure 7:**
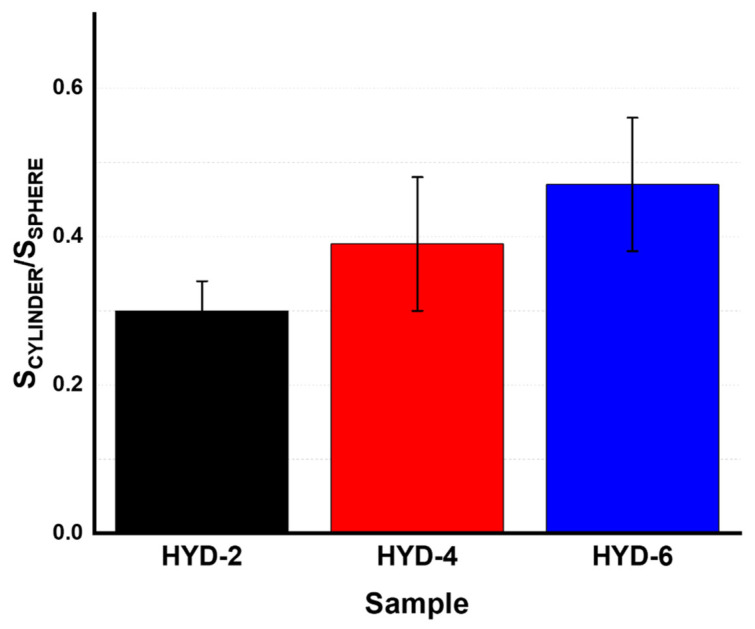
Relative swelling (S_CYL_/S_SPH_) for three representative temperatures: 10, 37 and 55 °C in PBS of each type of sample with an inner diameter of 6 mm.

The influence of geometry, i.e., between cylinders and spheres, can be established using, for instance, the 6 mm diameter systems (see Figure 7). Although both swell less at higher crosslinking, the spheres presented larger swelling capacities in comparison to the cylinders in all three cases (see the S_CYL_/S_SPH_ ratio, which is less than one in all three cases shown). This influence of the geometry, as well as the diameter, on the swelling follows the classical Gurney–Lurie plots for non-steady-state heat or mass transfer for simple 3D finite geometries, such as flat, cylinders and spheres [49,50]. In particular, the delay in diffusion is higher in flat hydrogels, where the larger surface area relative to volume contributes to a slower diffusion rate. Subsequently, the cylindrical hydrogels show a higher diffusion rate than flat hydrogels but exhibit more resistance to solvent diffusion than spherical systems. Likewise, spherical hydrogels exhibit higher diffusion rates, demonstrating that shape plays a fundamental role in the diffusion phenomenon. In addition, this incomplete diffusion is also dimension dependent, being larger for larger distances from the surface thickness of the hydrogel, also implying the influence of thickness on the aforementioned diffusion phenomena. The theoretical calculation results in a higher mass transfer of about 20% for the sphere compared to the cylinders after 24 h of the swelling experiments (note that measurements are made every 24 h). A detailed explanation of the calculations made is provided in the Supplementary Information. Finally, it seems that crosslinking also affects diffusion kinetics. In this sense, it is possible to observe that as the crosslinking content increases, it seems that the differences between the two structures in terms of swelling kinetics become smaller. Based on what has been observed in this manuscript, systems with a higher number of knots reduce their swelling capacity, which may explain the observable differences in terms of swelling kinetics.

**Figure 8:**
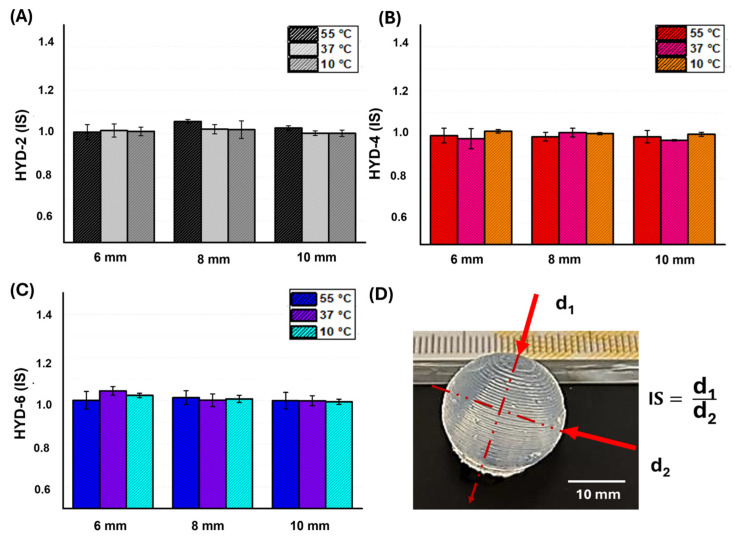
IS ratio (d_1_/d_2_) in PBS of the spheres at different temperatures (10, 37 and 55 °C) and different molar percent crosslinking. (**A**) HYD-2 (represented by black and close colors), (**B**) HYD-4 represented by red and close colors), (**C**) HYD-6 (represented by blue and close colors). (**D**) IS equation and a simple IS image measurement scheme over a spherical HYD-2 hydrogel where the arrows indicate the diameters evaluated (10 mm).

Complementarily, to analyze whether the hydrogel swells homogeneously in all spatial axes, IS evaluation of the spheres at different temperatures (10, 37 and 55 °C) were considered and shown in Figure 8. With the manufacturing technology described, all samples show an IS value close to value 1, indicating the isotropic swelling behavior of the hydrogels across the entire temperature range.

#### 2.3.2. Towards Complex Hydrogel Geometries: Preparation of Hydrogels from Basic Tubular Geometries to the Formation of Complex Stents

Once the fabrication of solid 3D hydrogels and their thermosensitivity have been analyzed, the formation of other complex hollow structures, such as tubes, was considered at this point to study as the next step. The proposed tubes, with an outer diameter of 10 mm, were again evaluated in terms of crosslinking percentage (HYD 2, 4 and 6) and wall thickness (1, 2 and 3 mm). All samples were again correctly manufactured as shown in Table 3. As in Table 2, variations in terms of swelling capacity and opacity can be observed, i.e., the increased crosslinking of the systems reduces the variation in dimensions and increases the opacity previously described. Similarly, the images also show qualitative differences in terms of opacity and swelling capacity with increasing wall thickness. Thus, as the wall thickness increases, the opacity of the system increases, and the absorption capacity of the hydrogels decreases. In short, the behavior observed in solid systems is also true for hollow structures.

**Figure 9:**
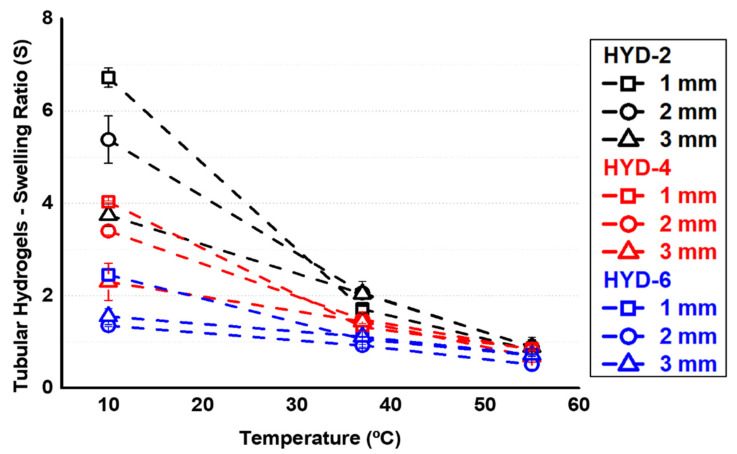
Swelling ratio in PBS at different thicknesses (1, 2 and 3 mm) of pVCL tubes for three representative temperatures: 10, 37 and 55 °C. Outer diameter of 10 mm.

As has been observed in a qualitative way in Table 3, Figure 9 shows the gravimetric studies for the different tubes proposed. Again, in agreement with the previous discussion for cylinders and spheres, while thermosensitivity was observed in all the analyzed samples, a clear decrease in the amplitude of the response was observed as the crosslinker content increased. Similarly, as the tube wall thickness increased, the hydrogels showed delays in terms of absorption, which also coincides with what was discussed above.

**Figure 10:**
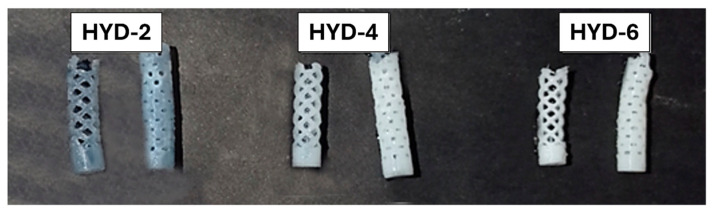
Image of different stents with the three molar percentages of crosslinking used (HYD-2, 4 and 6) suggesting the possibility of the formation of systems of more complex geometry based on the described technology.

Finally, to evidence the potential of this approach Figure 10 shows two complex hollow systems developed from the mold-based 3D printed approach described above. As can be seen, two different stent geometries with different percentages of crosslinking were prepared to prove the versatility of PVA sacrificial mold options in terms of design for the formation of networks with versatile tailor-made geometry.

### 2.4. Evaluation of Cytocompatibility and Cell Detachment Capability of Non-Planar Cultures from Curved T-Sensitive Hydrogels

**Figure 11:**
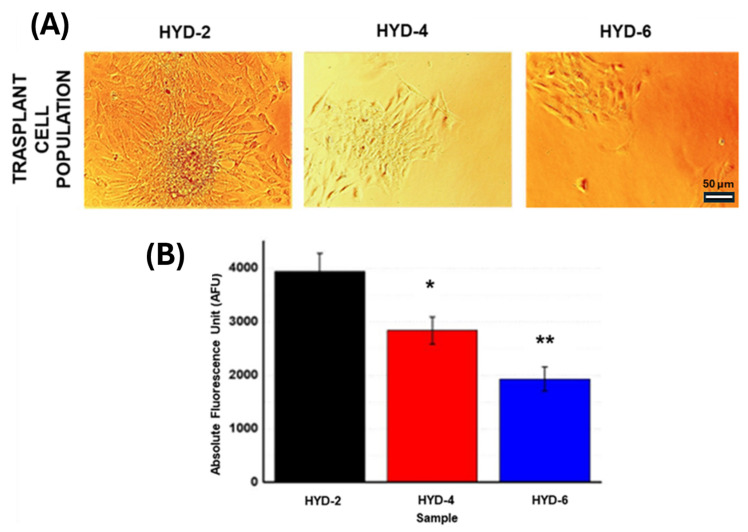
**(A**) Bright field images of C166-GFP cell culture 24 h after transplant from flat hydrogels (HYD-2, 4 and 6; white scale bar: 50 µm). (**B**) Metabolic activity quantification (Alamar Blue) of cell culture transplants. Significant differences were indicated as follows: * (*p* ≤ 0.01) and ** (*p* ≤ 0.01).

Thermosensitive VCL hydrogels synthesized with conventional methods present cell adhesion and proliferation capacity at a temperature of 37 °C and non-aggressive cell detachment capacity with a temperature decrease to values below the transition temperature, VPTT [23,24,25,26,27]. In this sense, cell detachment is directed by changes at the molecular level where the polymeric chains modulate their interaction with the medium depending on the external temperature (hydrophobic–hydrophilic, above or below the transition value, VPTT, respectively) [51]. Thus, in order to ensure cytocompatibility and verify the influence of the crosslinking percentage (and, therefore, the decrease in thermosensitivity of the systems HYD-2, 4 and 6) on the cell detachment capacity, flat hydrogels (0.5 mm) were employed as supports using an endothelial autofluorescent model (C166-GFP cell line) and incubated for 48 h. Despite the increased crosslinking density of the systems, all samples allowed for cell adhesion and proliferation. Next, to achieve cell transplantation through a controlled temperature decrease, flat hydrogels were turned upside down and placed into a new treated well, as described in the experimental section. However, based on images from Figure 11A, samples with higher crosslink percentages evidenced a significant reduction in their cell detachment capacity. This reduction in terms of cell population observed in the images was further analyzed by the quantitative analysis carried out with Alamar Blue (Figure 11B). In agreement with the images, the Alamar Blue test shows significant differences of the less crosslinked sample with respect to molar percentages of crosslinking 4 and 6. In summary, the need for a certain thermosensitive capacity to produce cell sheet detachment with this technology seems clear as a requirement [51] (see Supplementary Figure S4), where the swelling of HYD 2, 4 and 6 in PBS and 0.5 mm thickness is plotted over the entire temperature range. This situation indicates that hydrogels that barely show thermosensitivity (HYD-4 and 6) were not optimal for this objective but confirmed that HYD-2 thermosensitive networks are useful as smart supports for cell manipulation. For this reason, the system selected for conceptual testing in terms of non-planar cell monolayer detachment is the minimally crosslinked system (2 mol% or HYD-2). This hydrogel has robust mechanical characteristics that allow handling without compromising its integrity (169 ± 6 KPa elastic modulus in hydrated state for dynamic compression studies [32]). It has also been additionally studied with the premioblastic (C2C12-GFP; ATTC) and fibroblasts (SWISS-3T3; ATCC) cell lines in Figure S5 to demonstrate cell adhesion and proliferation capacity at 37 °C and culture detachment obtained in a non-aggressive way by controlled temperature decrease (15–20 °C) with other cell lines.

**Figure 12:**
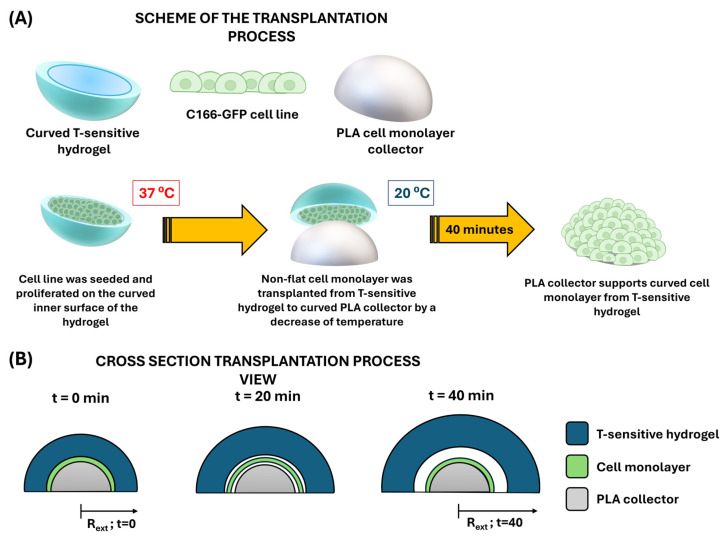
(**A**) Basic scheme for the transplantation of curved cell monolayers: the curved T-sensitive hydrogel allows cell adhesion and proliferation of endothelial cell lines at 37 °C; next, a printed PLA support with a complementary shape to the hydrogel is placed as a base and finally; a temperature decrease to approximately 20 °C causes a structural change of the network from a hydrophobic to a hydrophilic behavior that allows cell detachment to the printed target support. (**B**) Cross-section view of the transplantation process.

As already described, the limitations in terms of hydrogel design described in previous works [32] are solved with the sacrificial mold technology presented in this work. Thus, hydrogels with complex geometry can be obtained and at the same time act as intelligent non-planar supports for cell culture manipulation. As direct application, the present manuscript presents the synthesis of curved smart hydrogels for the transplantation of concave cell monolayers into a recipient (3D printed in PLA as a biocompatible support) that simulates the repair of semi-spherical tissue (inspired by the shapes of the cornea or articular cartilage), and thus will serve us to illustrate the versatility of the presented technology. As a summary, Figure 12A shows the scheme of the procedure. The process begins with the seeding of curved thermosensitive hydrogels obtained from PVA molds on the inner face, which will be the future transplant donor. After cell proliferation and monolayer formation at 37 °C, the hydrogel is placed in a complementary 3D printed prefabricated PLA cell collector. As mentioned above, by lowering the temperature (20 °C), the rapid hydration of the smart support and consequent swelling (see again Figure 12B) and associated network radius increase allows cell detachment, conserving extracellular matrix (ECM) and cell junctions during the process.

**Figure 13:**
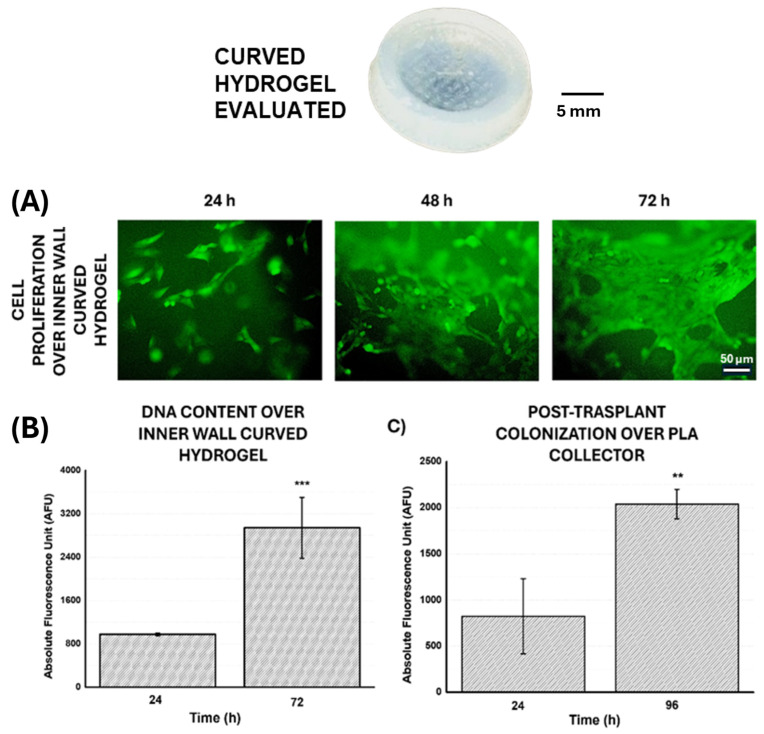
The curved hydrogel tested is attached at the top of the image (black scale bar: 5 mm). (**A**) Fluorescence images of C166-GFP culture of the inner wall of the curved hydrogel at 24, 48 and 72 h (white scale bar: 50 µm) and (**B**) DNA content study of cell proliferation also on the inner wall hydrogel at 24 and 72 h. (**C**) Metabolic activity (Alamar Blue) of cell transplants on PLA collector at 24 and 96 h. Significant differences were indicated as follows: ** (*p* ≤ 0.01) and *** (*p* ≤ 0.001).

To carry out this novel application, curved hydrogels obtained from PVA molds based on HYD-2 formulation were seeded with the C166-GFP autofluorescent cell line. Figure 13A shows the process of cell proliferation during 72 h on the inner surface of the hydrogel, demonstrating that the curved systems allow for cell adhesion and growth to confluence, forming a monolayer over the inner hydrogel surfaces. Quantitatively, as shown in Figure 13B, this growth was confirmed by the significant increase in DNA content observed from 24 to 72 h. After that, and based on the described methodology (see again schematic in Figure 12), the curved thermosensitive hydrogel was coupled to the printed PLA collector. Then, the system was subjected to a temperature decrease towards values below the VPTT that favors the detachment of the non-planar cell culture. In this way, the medium absorption by the hydrogel was promoted and with it an increase in its dimensions, conditions that become essential for the proposed objective of cell detachment [34] (see also Supplementary Figure S6 to observe the variation in diameter of the curved polymeric network as a function of temperature). After transplant, the quantitative study shown in Figure 13C analyses the metabolic activity of the culture on the PLA collector 24 and 96 h after the transplantation process. The significant increase in terms of metabolic activity analyzed assures the viability of the non-planar culture after the transplantation process.

Based on the previous description, the new process of obtaining hydrogels with complex geometry opens a wide field of study for the development of more precise and customizable therapies for cell sheet engineering technologies. Our methodology using thermosensitive hydrogels with complex geometries not only allows for an efficient detachment of non-planar culture with a controlled decrease in temperature, but also enables the development of more complex cellular constructs, already described in literature [52]. For example, in the corneal epithelium regeneration model, this technology could be used to optimize cell therapy applications and therefore tissue healing [53].

## 3. Conclusions

In this study, 3D VCL-based thermosensitive hydrogels with different geometries. i.e., cylinders, spheres, tubes and stents, and three molar crosslinking percentages (2, 4 and 6 mol%) have been successfully obtained by photocuring technology inside two-layer printed PVA sacrificial molds in water. The higher the crosslinking degree, the higher the shape fidelity with respect to the original mold due to the decrease in swelling capacity. The inherent roughness generated in the mold after the use of MatEx printing technology in the *z*-axis allows for obtaining hydrogels with surface roughness in the micrometer range which could be very interesting in modulating the interaction with cell cultures. A swelling and thermosensitivity decrease with increasing thickness has been observed, which has been related to a delay in terms of medium diffusion complying with the Gurney–Lurie theory of mass transfer for basic 3D geometries. Curved hydrogels with a 2 mol % of crosslinking degree, which have shown the highest cell detachment efficiency upon temperature decrease in control planar hydrogels as compared to more crosslinked samples, have been successfully evaluated as curved supports for cell culture and detachment. In this sense, the versatility of the technology presented in this work may facilitate the production of tailor-made hydrogels that allow more precise manipulation of non-planar cultures that could be very interesting in the field of cell sheet engineering.

## 4. Materials and Methods

### 4.1. Materials

N-Vinyl caprolactam (VCL), ethylene glycol dimethacrylate (EGDMA) and 1-hydroxyl cyclohexyl phenyl ketone (HCPK) were purchased from Sigma-Aldrich (St. Louis, MO, USA). 1,3-Divinylimidazolidin-2-one (DVI) was supplied by BASF. Absolute ethanol (EtOH) was purchased from Scharlau.

Poly vinyl alcohol (PVA), employed for the preparation of the sacrificial molds was purchased by KURARAY POVAL™ (Frankfurt, Germany) MOWLIFLEX^®^ (1.75 mm diameter filament). PLA employed to print the cell collector was purchased from Fillamentum Addi(c)tiven (1.75 mm filament). For both filaments, pieces were designed using the solid parametric modelling software Autodesk Inventor 3D 2023 and printed using MatEx technology on the Raise3D Pro2 3D printer with IdeaMaker software 4.3.3. as slicer assistant. The diameter of the nozzle used for both cases was 0.4 mm. The printing conditions for both materials (PVA and PLA) can be summarized as follows: 30 mm/s printing speed with a bed layer at 60 °C and 200 µm of layer height. In addition, fan speed was fixed at 100%. Finally, for extrusion temperature parameter, the temperature was fixed at 190 and 220 °C for PVA and PLA, respectively.

The cell line with fluorescent protein C166-GFP (CRL-1772) was purchased by ATTC. Fetal Serum Bovine (FBS) was purchased from Thermo Scientific (Hyclone^®^, Thermo Scientific, Waltham, MA, USA). Dulbecco’s Modified Eagle Medium (DMEM) and the antibiotic (penicillin, streptomycin and G418) were also supplied by Sigma-Aldrich. 12 and 24 well culture plates (treated and untreated, respectively) were purchased from Corning Costar (New York, NY, USA).

### 4.2. Synthesis of the Hydrogels

The hydrogels were synthesized by a one-step reaction from a conventional radical photopolymerization. To carry out the polymerization, a solution of VCL/EtOH (2.26 g/mL) was mixed with 0.5 wt.% of the photoinitiator HCPK. In the same way, two crosslinkers EGDMA and DVl were mixed with a molar ratio of 80/20. Throughout the present study, different crosslinking percentages were prepared in the formulation (2 as a control, 4 and 6 mol%) to analyze their influence on the physicochemical properties of the obtained networks. The prepared mixtures were bubbled with N_2_ to remove confined oxygen and transferred to different PVA molds using a syringe. For the preparation of flat hydrogels, PVA molds were separated by commercial silicone as spacers. Finally, photopolymerization was carried out inside a UV chamber with five lamps generating data close to 3500 μW/cm^2^ (model CL-1000 L, 230 V), for 40 min and λ = 365 nm. In the case of the comparative study between the transparent control molds and the PVA-based sacrificial mold used in this work, it is necessary to mention that the control mold was made of polystyrene sheets coated with low density polyethylene films (PS/LDPS).

After photopolymerization, the hydrogels were recovered from the PVA molds with successive distilled water (diH_2_O) washes until all residual mold and any remaining unpolymerized elements were removed. The samples were stored in EtOH/diH_2_O (70%). 24 h before the different experiments, the samples were transferred back to diH_2_O and washed several times to completely the removal of EtOH.

### 4.3. Characterization of the Hydrogels

Chemical characterization of networks by spectroscopy (FTIR-ATR) measurements were carried out on a PerkinElmer Spectrum One spectrometer (PerkinElmer Life and Analytical Sciences Inc., Wellesley, MA, USA) using an ATR device in the range of 400–4000 cm^−1^ with a resolution of 2 cm^−1^.

Thermosensitive Evaluation of the Hydrogels

The swelling ratio (S) is defined as the weight of water absorbed by the network with respect to the weight of the polymeric network in the dry state, see Equation (3). In order to evaluate the swelling properties of the hydrogels, samples were measured every 24 h for each temperature and condition. Finally, for the case of flat hydrogels, networks were die-cut in the swollen state at room temperature (20 °C) in a circular shape with a diameter of 12 mm.

(3) $$S=\frac{\left(W_{s}-W_{d}\right)}{W_{d}};$$

where W_s_ is the weight of the hydrogel in the swollen state and W_d_ is the weight of the dried hydrogel. Measures were evaluated by triplicate in PBS. Relative data (S/S) were represented considering propagation of errors.

In the case of sphere evaluation, their isomorphic swelling (IS) was analyzed as the ratio between the orthogonal diameters of a sphere (see Equation (4)). Diameters were measured every 24 h for each temperature and condition. Thus, when the ratio result is close to 1, the swelling capacity of the spheres is homogeneous and, therefore, isomorphic in all directions.

(4) $$IS=\frac{Diameter(1)}{Diameter(2)};$$

On the other hand, to analyze the variation of transmittance of the samples as a function of temperature and degree of crosslinking, the UV-Vis SPECORD 205 spectrophotometer (Headquarters, Santa Clara, CA, USA) associated with WinASPECT Plus 2.2 software was used at visible wavelength (600 nm) at different temperatures (20 and 37 °C). Hydrogels were stabilized in PBS at the appropriate temperature 24 h before the assay to ensure total polymer–media equilibrium. Measures were carried out by triplicate. In the case of evaluating the transmittance of the prepared PVA molds with different numbers of layers, films with dimensions to fit in spectrophotometer cuvettes were printed and measured a UV wavelength of 365 nm (it is worth mentioning that this wavelength value will be later employed for the photopolymerization process).

Finally, regarding the surface roughness of the hydrogel in the *z*-axis and of the 3D printed parts of PVA, the images shown were taken using a Leica/S6D stereo microscope equipped with a Leica DFC320 camera associated with Leica LAS software (1.4.5). In addition, a Zeta Instruments 3D optical profiler, model Zeta-20, was used for a more precise study. The quantification of the images was subsequently performed using the free software “profilmonline.com, accessed on 1 June 2024)” [54].

### 4.4. Biological Evaluation

#### 4.4.1. Hydrogel Preparation for Cytocompatibility Evaluation

First, flat hydrogel discs of 16 mm diameter (to fit in a 24-well culture plate) were die-cut in PBS (Phosphate Buffered Saline, pH 7.4) at 37 °C to be used as a control material. After that, both flat hydrogel discs and free form curved hydrogels obtained from PVA molds were sterilized by washing with EtOH at 70% for 10 min three times. Later, hydrogels were also extensively washed three times with 2 mL in PBS. Then, samples were irradiated by UV light for 20 min for each side. Then, after a last wash step with DMEM (Dulbeco’s Modified Eagle Medium) high in glucose (D6429), samples were left in DMEM supplemented with 1% of antibiotics (PS, 100 U/mL penicillin) and 10% of FBS (Fetal Bovine Serum) overnight in an incubator at 37 °C and 5% of CO_2_. Finally, it should be noted that the PLA 3D printed collector was sterilized in the same way as the hydrogels without the aforementioned initial PBS washes.

#### 4.4.2. Cell Culture

A C166-GFP mouse endothelial cell line was used to assess the cytocompatibility of the hydrogels studied. This cell line expresses GFP (Green Fluorescent Protein) genes to facilitate cell monitoring studies. The cell line was cultured with DMEM supplemented in an incubator at 37 °C and 5% CO_2_ and cell passages were performed when 90% confluence was reached. Flat hydrogels were seeded at a density of 2 × 10^4^ cells/cm^2^ and allowed to proliferate for 48 h in an incubator (also 37 °C and 5% of CO_2_). In the case of curved hydrogels, the cell line was homogeneously seeded at a density of 5 × 10^4^ cells/cm^2^ and allowed to proliferate for 72 h in an incubator with the same conditions as those used in flat samples. Both studies were monitorized with an inverted fluorescence microscope (Olympus IX51, Olympus Evident, Barcelona, Spain) with FITC filter (λ_ex_/λ_em_ 590/530 nm) at 10× magnification.

#### 4.4.3. Cell Sheet Detachment by Controlled Decrease of Temperature

In order to carry out the cell detachment process by temperature decrease applied for flat type hydrogels after 48 h of proliferation, the cultures obtained were transplanted into a new treated culture plate (TCP). First, 70% of the final volume of the medium (5–10 °C) was added to the new culture plate to keep the hydrogel side in contact with the cell culture hydrated. The hydrogels were then turned over and placed on the new plate for 20 min at 15–20 °C to cause detachment. After 20 min, the remaining 30% of the medium was added to complete the 40-min transplantation process. Finally, the hydrogels were removed and the transplanted cultures were re-incubated (37 °C and 5% CO_2_).

In the case of the curved systems, after 72 h of proliferation, the culture obtained was also transplanted by lowering the temperature; this time to the 3D printed PLA cell collector, also in a new TCP (see Figure 12A). For this case, 1 mL of medium at 5–10 °C was added again to keep the hydrogel surface with the matured culture hydrated. Subsequently, the hydrogel with the cell side was placed on the printed cell collector and kept at 15–20 °C for 40 min to complete the transplantation. It is worth mentioning that after 20 min of testing, another ml was added to complete the required the required medium volume. Finally, the curved hydrogel was removed and the collectors with the transplanted cultures were placed in incubation (37 °C and 5% CO_2_).

#### 4.4.4. Cell Viability Analysis Metabolic Activity and dsDNA Quantitation

Metabolic activity of the cell cultures in the manuscript were quantified with Al-amar blue assay (Biosource, San Diego, CA, USA). Following the manufacturer’s instructions, it was added 10% of the indicated reagent to the target culture and incubated for 90 min. Subsequently, fluorescence readings taken in triplicate (λ_ex_/λ_em_ 535/590 nm) were obtained using a multiwell plate reader (Synergy HT, BioTek, Winooski, VT, USA).

In turn, the dsDNA content of the cell culture was measured using the Flu-oReporter™ Blue dsDNA fluorometric quantification kit (Thermofisher, Waltham, MA, USA). The protocol applied can be summarized as follows: washing with distilled water and incubation at 37 °C for 1 h, subsequent freezing of the culture at −80 °C and finally staining with Hoechst 33,258 reagent. Quantification was performed by Fluorescence (λ_ex_/λ_em_ 360/460 nm). It should be noted that measurements were performed in triplicate using a plate reader (Synergy HT, Brotek, Mehsana, India).

#### 4.4.5. Statistical Analysis

The results were compared to evidence of the differences between hydrogels. Statistical analysis was calculated using the Student *t*-Test with Graph Pad 4 software 10.4.0. Significant differences represent: *(*p* ≤ 0.05), ** (*p* ≤ 0.01), *** (*p* ≤ 0.001).

## Figures and Tables

**Table 1:** Surface roughness data generated in the *z*-axis (µm) after the use of sacrificial molds for two temperatures (20 and 55 °C). The figure above provides a basic schematic of the *z*-axis wrinkling surface process and images of HYD-2, 4 and 6 taken with the microscope (scale bar: 5 mm).

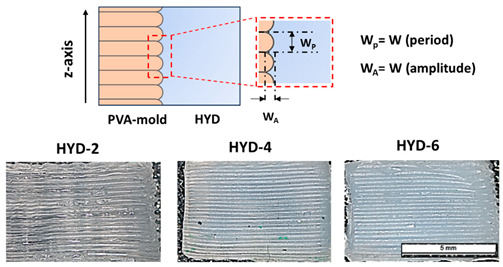				
**Sample/Label**	**Surface Roughness (µm)**			
	**Wrinkle Period at (°C)**		**Wrinkle Amplitude at (°C)**	
	**20**	**55**	**20**	**55**
HYD-2	282 ± 42	203 ± 17	868 ± 90	290 ± 90
HYD-4	242 ± 23	203 ± 23	743 ± 113	270 ± 53
HYD-6	230 ± 16	207 ± 15	577 ± 77	327 ± 77

**Table 2:** Images of all types of cylinders and spheres prepared in this work.

Scale Bar: 10 mm	Cylinders						
Mold inner Diameter (mm)	2		4		6		8
HYD-2	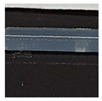		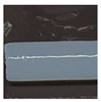		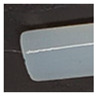		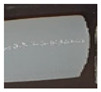
HYD-4	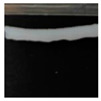		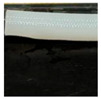		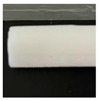		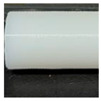
HYD-6	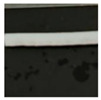		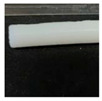		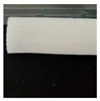		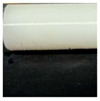
**Spheres**							
Mold inner Diameter (mm)		6		8		10	
HYD-2		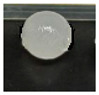		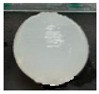		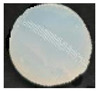	
HYD-4		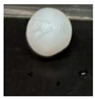		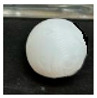		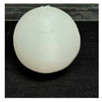	
HYD-6		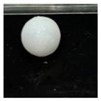		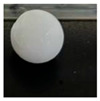		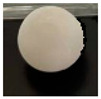	

**Table 3:** Images of all types of tubes (outside diameter of 10 mm) prepared in this work.

Scale Bar: 10 mm	Tubes		
Wall Thickness (mm)	1	2	3
HYD-2	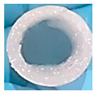	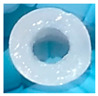	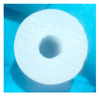
HYD-4	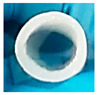	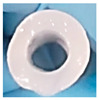	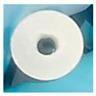
HYD-6	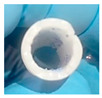	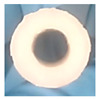	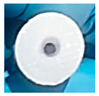

## Data Availability

The original contributions presented in this study are included in the article/Supplementary Material. Further inquiries can be directed to the corresponding authors.

## Supplementary Materials

The following supporting information can be downloaded at: https://www.mdpi.com/article/10.3390/gels10120802/s1, Figure S1: Comparative FTIR analysis of the hydrogels manufactured with two types of mold: PS conventional and two-layer PVA sacrificial mold; Figure S2: Fourier transform infrared (FTIR) spectroscopy of the flat hydrogels synthetized with a different crosslinking molar percentage (2, 4 and 6), represented by the colors black, red and blue, respectively; Figure S3: Surface roughness of HYD-2, 4 and 6 along the axis z for two temperatures evaluated (25 and 55 °C). Figure S4: Thermosensitive evaluation in PBS for flat hydrogels (HYD-2, 4 and 6) with 0.5 mm of thickness. Swelling Ratio (S) in the range of 10 to 55 °C and VPTT value, where HYD-2, 4 and 6 were represented by the colors black, blue and red, respectively; Figure S5: Cell growth images (24 and 144 h) of the transplants on flat PLA-printed support for the different cell lines (C166-GFP, C2C12-GFP and SWISS-3T3) together with quantitative analysis based on Alamar Blue reactive; (white scale bar: 200 µm). Figure S6: Variation of curved T-sensitive hydrogel diameter in mm as a function of temperature. In this document of Supplementary Materials it is also possible to find the calculations associated with the Gurney–Lurie theory.
