# Supplementary material for: Development of Thermosensitive Hydrogels with Tailor-Made Geometries to Modulate Cell Harvesting of Non-Flat Cell Cultures
Source: Gels. 2024 Dec 6;10(12):802. doi: 10.3390/gels10120802 (PMC11675774; doi:10.3390/gels10120802)
Supplement: Supplementary file 1 [file gels-10-00802-s001.zip › gels-3283861-supplementary.pdf]

# Supplementary Material

## Development of thermosensitive hydrogels with tailor-made geometries to modulate cell harvesting of non-flat cell cultures.

Rubén García-Sobrino [1, 2, 3*], Enrique Martínez-Campos [1, 2], Daniel Marcos-Ríos [1, 2], Zenen Zepeda-Rodríguez [4], Juan L. Valentín [4], Raúl Sanz-Horta [1], Marina León-Calero [1], Helmut Reinecke [1], Carlos Elvira [1], Alberto Gallardo [1], Juan Rodríguez-Hernández [1*],

[1] Polymer Functionalization Group. Instituto de Ciencia y Tecnología de Polímeros-Consejo Superior de Investigaciones Científicas (ICTP-CSIC), Departamento de Química Macromolecular Aplicada. Calle Juan de la Cierva, nº 3, 28006 Madrid, Spain.

[2] Group of Organic Synthesis and Bioevaluation, Instituto Pluridisciplinar, Universidad Complutense de Madrid (UCM). Associated Unit to the ICTP-IQM-CSIC. Paseo Juan XXIII, nº 1, 28040 Madrid, Spain.

[3] Department of Applied Mathematics, Materials Science and Engineering and Electronic Technology, Universidad Rey Juan Carlos, Calle Tulipán s/n, 28933 Móstoles, Spain.

[4] Elastomers Group. Instituto de Ciencia y Tecnología de Polímeros-Consejo Superior de Investigaciones Científicas (ICTP-CSIC). Calle Juan de la Cierva, nº 3, 28006 Madrid, Spain.

* Corresponding author: ruben.sobrino@urjc.es & jrodriguez@ictp.csic.es

Tel.: (+ 34 91 562 29 00).

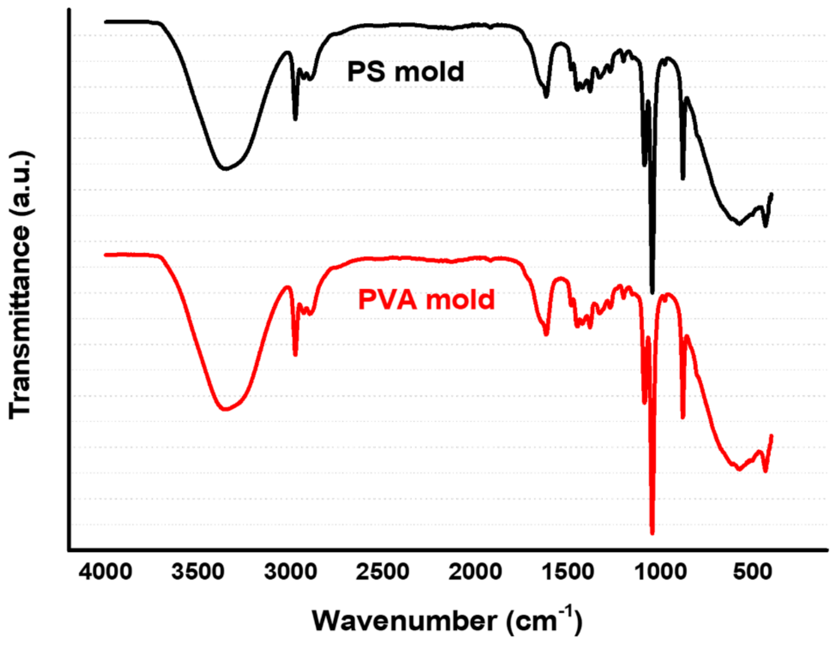


**Figure S1.** Comparative FTIR analysis of the hydrogels manufactured with two types of moulds: PS conventional and two-layer PVA sacrificial mould.

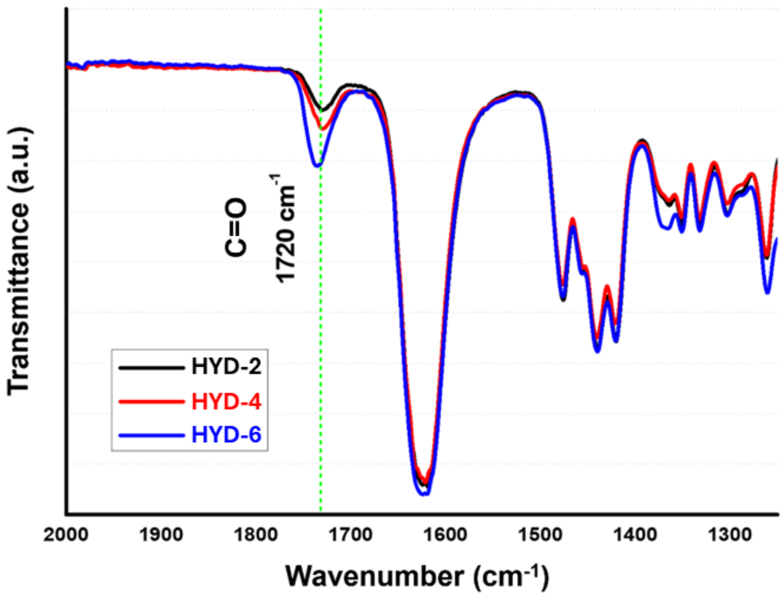


**Figure S2.** Fourier transform infrared (FTIR) spectroscopy of the flat hydrogels synthetized with a different crosslinking molar percentage (2, 4 and 6), represented by the colours black, red and blue, respectively.

## Gurney-Lurie theory

The Gurney-Lurie theory, as well as the corresponding charts, provides a solution to the problem of heat or mass transfer in a non-steady state for finite 3D geometries, such as cylinders and spheres. In order to apply Gurney-Lurie plots, it is necessary to express certain parameters: change in concentration not reached (Y) and relative time ($X_D$).

$$Y = \frac{CA_S - CA}{CA_S - CA_0}; \text{ (S1)}$$

$$X_D = \frac{D_{AB}t}{x_1^2}; \text{ (S2)}$$

In this case of Eqn (S1), $CA_S$ is the superficial concentration of solvent in the samples during the swelling experiments, $CA_0$ is the initial concentration of solvent in the particle and CA is defined as the concentration in the sample at a determined position and time. For the case of Eqn (S2), $D_{AB}$ is the diffusion mass transfer coefficient t is the time and $x_l$ is the characteristic length from the point of symmetry (radius in the case of cylinders and spheres of this manuscript). In addition to the existence of a transient diffusive transition process as mentioned above, it is an essential requirement to consider that $D_{AB}$ and $CA_0$ are constant and uniform during the process and that the applicable limits are also constant and uniform. At the center of both geometries, the relative position value and the relative resistance value are neglected because there is no convection resistance and molecular diffusion controls the flux of diffusing species in the process. For convenience, mass transfer was also assumed to occur only in the radial position of the cylinders and spheres.

To apply equations S1 and S2, it must be taken into account that $CA_0=0$ at the beginning of the experiment since we start from dry hydrogel. We may define $CA_S=1$ (water concentration at the surface) and CA=0.999 (the concentration at the end of the experiment or equilibrium). Assuming the same $D_{AB}$ for all geometries, that all samples were subjected to the same time (t=24 h) and that all had the same characteristic length (diameter) ($x_l=0.3$ cm), we can go to the Gurney Lurie charts with a common value on the X-axis. For similar X, the Y-axis difference (concentration not reached) is about 20% (higher concentration of 20% for the sphere compared with the cylinders at 24 hours of swelling experiments).

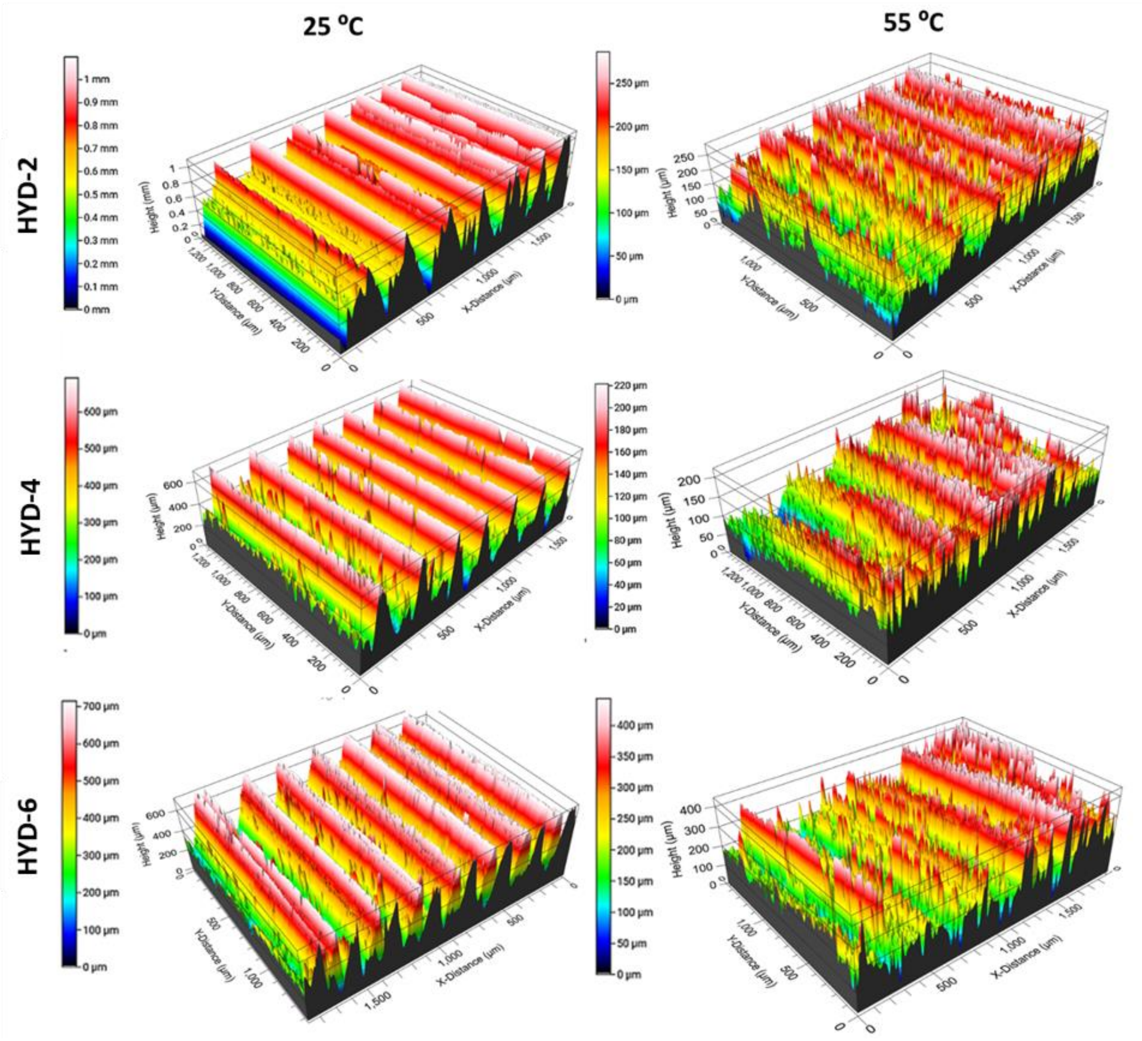


**Figure S3**. Surface roughness of HYD-2, 4 and 6 along the axis z for two temperatures evaluated (25 and 55 ºC).

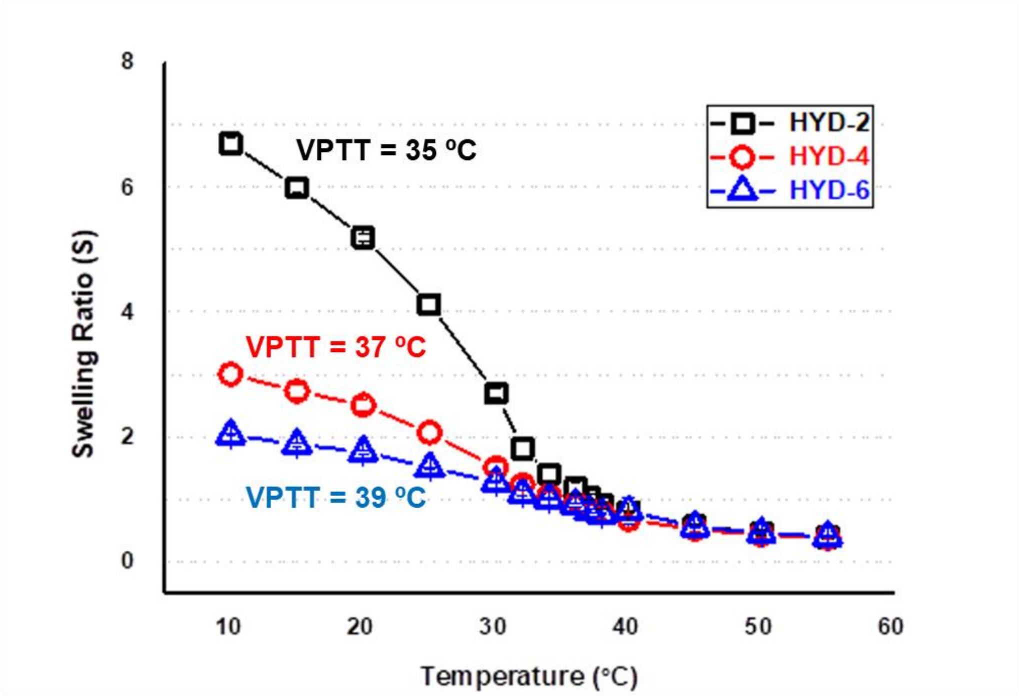


**Figure S4.** Thermosensitive evaluation in PBS for flat hydrogels (HYD-2, 4 and 6) with 0.5 mm of thickness. Swelling Ratio (S) in the range of 10 to 55 ºC and VPTT value, where HYD-2, 4 and 6 were represented by the colours black, blue and red, respectively.

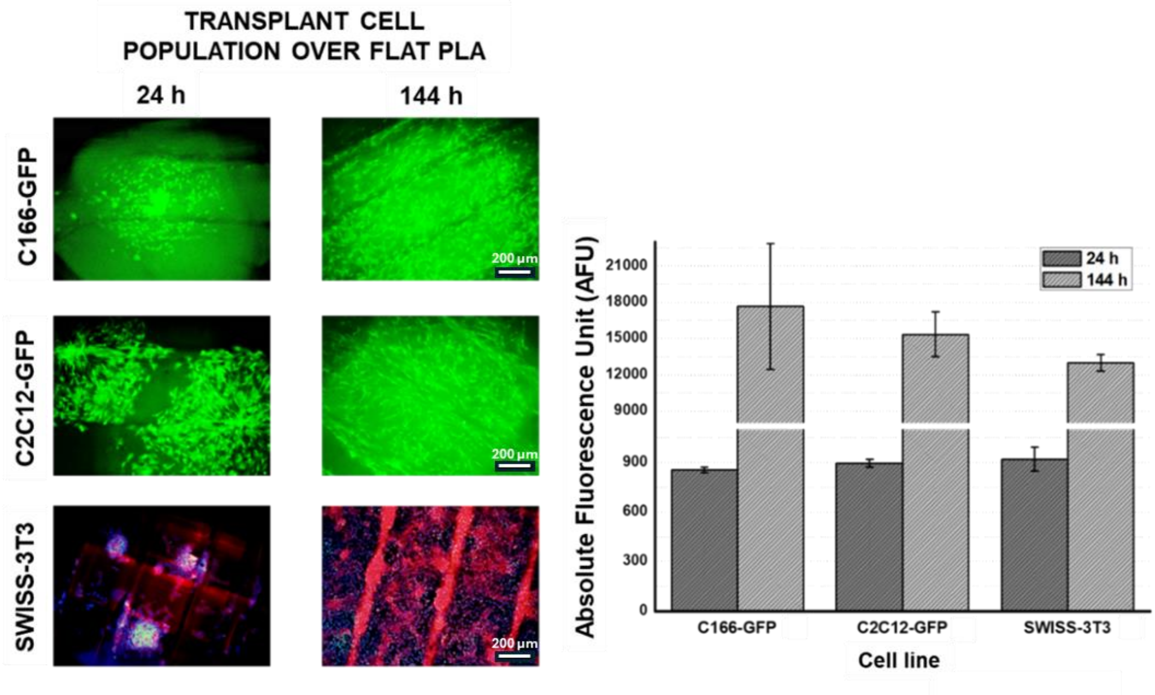


**Figure S5.** Cell growth images (24 and 144 h) of the transplants on flat PLA-printed support for the different cell lines (C166-GFP, C2C12-GFP and SWISS-3T3) together with quantitative analysis based on Alamar Blue reactive; (white scale bar: 200 µm).

Individually, and from flat systems, two cell lines in addition to the one presented in this work, i.e. C2C12-GFP and the fibroblast line SWISS-3T3, were proposed as a complementary study to demonstrate the efficiency of the aforementioned hydrogels to detach different cell lines. For this purpose, the hydrogels were seeded at a cell density of $1.5x10^4$ cells/cm$^2$ and left in incubation for 48 h (37 °C and 5 % $CO_2$). After this, a controlled temperature decrease was applied to motivate cell detachment to PLA-printed constructs. As a result, all cell lines showed detachment capacity and in turn cell growth capacity on the new substrate (24 and 48 h after the transplantation process) with similar growth times.

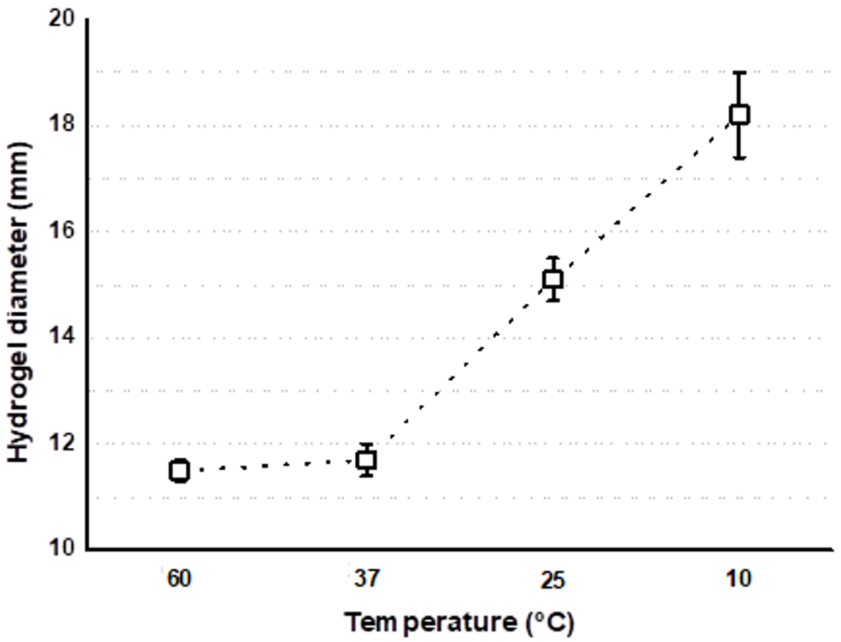


**Figure S6.** Variation of curved T-sensitive hydrogel diameter in mm as a function of temperature.
